# Comparative Genomics of Lactococcus spp. From Global Aquaculture Outbreaks Reveals Virulence Determinants, Antibiotic Resistance, and Phage Defence Mechanisms

**DOI:** 10.1002/mbo3.70147

**Published:** 2025-11-14

**Authors:** Adam M. Blanchard, Bailey Secker, Robert J. Atterbury, Samantha J. Windle, Ha Thanh Dong, Janchai Wongkaew, Le Thanh Dien, David Huchzermeyer, Bernard Mudenda Hang'ombe, Saengchan Senapin

**Affiliations:** ^1^ School of Veterinary Medicine and Science University of Nottingham Leicestershire UK; ^2^ Aquaculture and Aquatic Resources Management Program, School of Environment, Resources and Development, Klong Luang, Pathum Thani Thailand Asian Institute of Technology (AIT) Khlong Nueng Pathum Thani Thailand; ^3^ Faculty of Applied Technology, School of Technology Van Lang University Ho Chi Minh City Vietnam; ^4^ North West University Potchefstroom South Africa; ^5^ School of Veterinary Medicine University of Zambia Lusaka Zambia; ^6^ National Centre for Genetic Engineering and Biotechnology (BIOTEC) National Science and Technology Development Agency Khlong Nueng Pathum Thani Thailand; ^7^ Fish Health Platform, Centre of Excellence for Shrimp Molecular Biology and Biotechnology (Centex Shrimp), Faculty of Science Mahidol University Bangkok Thailand

**Keywords:** antimicrobial resistance, bacteriophage, fish, Lactococcus, prophage, whole genome sequencing, zoonotic

## Abstract

Lactococcosis is a major bacterial disease impacting rainbow trout production in South Africa and Southeast Asia, particularly during summer. In this study, 15 isolates from affected aquaculture facilities were characterised, revealing *Lactococcus petauri* (*n* = 12) as the predominant species, rather than the traditionally recognised *L. garvieae* (*n* = 3). This indicates a potential shift in the aetiology of lactococcosis with implications for diagnosis and management. Genomic screening identified multiple virulence factors, including adhesins in 14 isolates, capsular polysaccharide biosynthesis genes in 12, and sortase‐anchored proteins in all isolates, highlighting strain‐specific differences in pathogenic potential. Antimicrobial resistance (AMR) profiling revealed *ermB* (*n* = 10) and *tetS* (*n* = 11), consistent with resistance to macrolides and tetracyclines commonly applied in aquaculture. Phenotypic susceptibility testing against eight antimicrobial agents showed uniform resistance to nalidixic acid (15/15 isolates), alongside resistance to trimethoprim (12/15), sulfamethoxazole (11/15), and ciprofloxacin and oxacillin (7/15 each). These phenotypic results, while not fully aligned with the ARG profile, reflect aquaculture‐relevant antimicrobial exposures and indicate the presence of both intrinsic and acquired resistance mechanisms. Most (13/15) isolates contained 1–3 prophage regions, although none of these harboured any known virulence or AMR genes. However, they did genes encoding phage defence such as AbiD and R‐M systems. This information is important when considering the potential development of phage therapy to control piscine disease. Together, these findings advance understanding of the epidemiology, pathogenicity, and resistance dynamics of *Lactococcus* species in aquaculture and underscore the need for sustainable strategies to mitigate lactococcosis outbreaks.

## Graphical abstract

*Lactococcus petauri* predominated in isolates from outbreaks of lactococcosis, suggesting a shift in disease aetiology. Genomic analysis revealed diverse virulence factors, prophage and resistance genes, while antimicrobial susceptibility testing showed uniform resistance to common antibiotics found in farming systems, emphasising the need for sustainable control strategies in aquaculture.

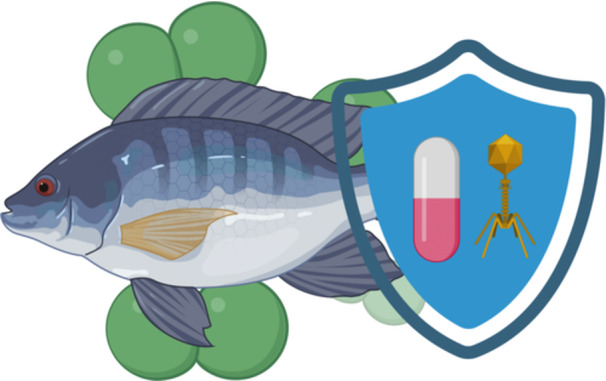

## 1 Introduction

Fish aquaculture is a rapidly growing sector of global food production, contributing significantly to food security, economic development, and livelihoods worldwide (The State of World Fisheries and Aquaculture 2024 2024). Among the diverse species farmed, economically important fish such as tilapia (*Oreochromis* spp.), rainbow trout (*Oncorhynchus mykiss*), and Asian seabass (*Lates calcarifer*) have gained prominence due to their high market demand, nutritional value, and adaptability to various aquaculture systems (Biology and Culture of Asian Seabass *Lates calcarifer* 2013; D'Agaro et al. 2022; Pullin 2025). Tilapia is a staple in both developing and developed countries, prized for its affordability and rapid growth rate. Rainbow trout is highly valued for its premium quality, appealing taste, and contribution to high‐value markets. Similarly, Asian seabass is a highly valued species in the aquaculture sector, known for its versatility in farming environments, excellent growth performance, and economic value in both domestic and export markets. The increasing reliance on fish aquaculture to meet the protein needs of a growing human population highlights its importance. However, the sustainability and productivity of the industry are increasingly threatened by bacterial infections (Lan et al. 2024; Duman et al. 2023; Soto et al. 2025), which result in significant economic losses and pose challenges to maintaining fish health and welfare.

There are several common bacterial diseases affecting farmed fish, including streptococcosis, aeromonasis, columnaris, edwardsiellosis and lactococcosis. Among these, lactococcosis—caused by lactococcosis‐causing bacteria (LCB)—is one of the emerging concerns in Africa and Southeast Asia. This disease affects a range of farmed fish, including tilapia and rainbow trout, resulting in economic losses for aquaculture operations (Heckman et al. 2024). The most common pathogen responsible for lactococcosis is *Lactococcus garvieae*, but other species such as *Lactococcus petauri* and *Lactococcus formosensis* have also been recently reported to contribute to the disease. Lactococcosis is typically characterized by clinical signs such as exophthalmos, acute haemorrhagic septicaemia, with mortality rates between 50% and 80% (Abu‐Elala et al. 2020). Lactococcosis is classed as an emerging global zoonotic pathogen with cases in marine and terrestrial mammals (Thiry et al. 2021; Evans et al. 2006), reptiles (Capobianco et al. 2024), birds (Barakat et al. 2000), and also rarely in humans (Colagrossi et al. 2022). Outbreaks have occurred globally in aquaculture systems (Savvidis et al. 2007; Meyburgh et al. 2017) which have a significant economic and welfare impact to the industry (Vendrell et al. 2006).

Outbreaks of disease are normally attributed to *L. garvieae*. However, classical microbiological identification techniques (Egger et al. 2023; Stoppani et al. 2023) often fail to differentiate this species from related aetiological agents such as *L. petauri* and *L. formosensis*. Additionally, pathogenesis is poorly defined and there is a lack of knowledge around virulence determinants in the three species, as most research is focused on clinical isolates from aquaculture (Gibello et al. 2016). Moreover, recent comparative genomics studies (Lin et al. 2023; Morita et al. 2011) have identified other homologous streptococcal virulence genes associated with adhesion (Miyauchi et al. 2012), iron transport (Schmidtke and Carson 2003) and capsular polysaccharide (Barnes et al. 2002).

Encapsulation has long been considered a virulence factor in many streptococcal species as it masks the cell surface antigens from the immune system (Gendrin et al. 2018; Shi et al. 2024). The capsule is immunogenic, and a target for antimicrobial drugs in many bacterial species. Shedding of the capsule on exposure to these compounds can be used as an evasion technique to evade autophagy‐mediated killing in macrophages (Kietzman et al. 2016). By comparing pathogenic and non‐pathogenic strains of piscine *L. garvieae* the presence of a capsule has been shown to be crucial for virulence in fish (Morita et al. 2011), however, it is sometimes not present, meaning encapsulation is not the sole requirement for virulence (Ture and Altinok 2016).

Adhesins are cell‐surface components that facilitate the binding of bacteria to host cell receptors. *L. garvieae* has been found to produce three proteins (PsaA, PavA, and enolase) with high similarity to adhesion virulence factors in other streptococcal species, as well as adhesin clusters 1 and 2 (*adhCI* and *adhCII*) and adhesin (*adh*) (Miyauchi et al. 2012; Ture and Altinok 2016). Sortase‐ anchored proteins contribute to the establishment and persistence of disease, and as such are likely to be virulence factors (Egan et al. 2010).

Antibiotics have long been deployed to control *Lactococcus* spp and *Streptococcus* spp. generally in aquaculture. Most commonly these include erythromycin, oxytetracycline, amoxicillin and low‐level doxycycline (Diler et al. 2002). However, resistance to erythromycin, oxytetracycline and lincomycin is growing due to the presence of *ermB* and *tetS (*Kawanishi et al. 2005 *)*. Outbreaks are becoming more frequent and control through medicated feed often fails due to a combination of inappetence and antimicrobial resistance (AMR) (Savvidis et al. 2007).

This study provides the first comparative genomic analysis of Lactococcus species isolated from lactococcosis outbreaks in aquaculture facilities across Zambia, South Africa, Vietnam, and Thailand. By characterising these isolates, we aimed to elucidate the genetic determinants underpinning AMR, virulence factors—including capsule formation, adhesion mechanisms, and phage resistance strategies and to evaluate their implications for effective disease management. Our findings offer crucial insights into pathogen evolution and highlight considerations for the future use of phage therapy as an alternative strategy to combat lactococcosis, ultimately contributing to improved sustainability and fish welfare in global aquaculture systems.

## 2 Methods

### 2.1 Bacterial Isolates

*Lactococcus* spp. isolates from Thailand (*n* = 3), Vietnam (*n* = 5), Zambia (*n* = 5), and South Africa (*n* = 2), previously obtained from diseased fish species including tilapia, Asian seabass, and rainbow trout were employed for whole genome sequencing in this study (Table 1). These isolates were previously identified as *L. garvieae* through 16S rDNA sequencing. Virulence test results from experimental infections using some of these isolates are also presented in Table 1 (Wongkaew et al. 2025).

**Table 1:** Provenance of the strains used in the study.

Origin	Date	Province	Species^a^	Host	ID	Virulence
Thailand	Oct 22	Kanchanaburi	*L. garvieae*	Red Tilapia	3749	No (Wongkaew et al. 2025)
Thailand	May 22	Chachoengsao	*L. petauri*	Asian sea bass	3750	No (Clinical and Laboratory Standards Institute 2020)
Thailand	May 22	Chachoengsao	*L. petauri*	Asian sea bass	3752	N/A
Zambia	Mar 17	Lake Kariba	*L. petauri*	Nile tilapia	3816	N/A
Zambia	Oct 17	Lake Kariba	*L. petauri*	Nile tilapia	3817	N/A
Zambia	Feb 18	Lake Kariba	*L. petauri*	Nile tilapia	3818	N/A
Zambia	Oct 18	Lake Kariba	*L. petauri*	Nile tilapia	3819	N/A
Zambia	Nov 18	Lake Kariba	*L. petauri*	Nile tilapia	3820	N/A
South Africa	Nov 15	Unknown	*L. petauri*	Rainbow trout	3821	N/A
South Africa	May 16	Unknown	*L. petauri*	Rainbow trout	3822	N/A
Vietnam	Aug 22	Ben Tre	*L. petauri*	Red Tilapia	3830	Low
Vietnam	Aug 22	Ben Tre	*L. garvieae*	Red Tilapia	3831	Medium
Vietnam	Aug 22	Ben Tre	*L. garvieae*	Red Tilapia	3832	High
Vietnam	Jan 22	Tien Giang	*L. petauri*	Red Tilapia	3833	Medium
Vietnam	Jan 22	Tien Giang	*L. petauri*	Red Tilapia	3834	Low

^a^ Species level taxonomic assignment of the 15 Lactococcus genomes is based on digital DNA:DNA hybridisation determined in this study.

### 2.2 Antimicrobial Susceptibility Test

Antimicrobial susceptibility was assessed using the standard disc diffusion method in accordance with CLSI guidelines (Clinical and Laboratory Standards Institute 2020) and previously published protocols (Sezgin et al. 2023; Perrin‐Guyomard et al. 2005). Antimicrobial discs (HiMedia, India) containing amoxicillin (AML) 30 µg, ciprofloxacin (CIP) 10 µg, erythromycin (ETM) 15 µg, nalidixic acid (NA) 30 µg, oxacillin (OX) 5 µg, oxytetracycline (OT) 30 µg, sulfamethoxazole (SX) 25 µg, and trimethoprim (TR) 30 µg were used. Briefly, isolates were grown in TSB and incubated at 30°C with shaking at 250 rpm for 24 h. The bacteria culture was then adjusted in a 0.85% NaCl (w/v) solution to obtain an optical density of 1 at 600 nm ( ~ 108 CFU/mL). The bacterial suspension was spread on Mueller Hinton Agar (Difco, BBL, USA) using a sterile cotton swab. After drying, antimicrobial discs were placed on the surface of the agar plates and incubated at 30°C for 24 h, after which the diameters of complete inhibition were measured and averaged from duplicate tests. *Staphylococcus aureus* 1020 was used as a control bacterial isolate. The number of resistant antibiotics divided by the total number of antibiotics tested was then used to calculate the multiple antibiotic resistance (MAR) index (Krumperman 1983). An empty sterile disc was used as a negative control.

### 2.3 DNA Extraction

DNA extraction of *Lactococcus* spp. was performed following previously optimised methods (Salvà Serra et al. 2018) with some modifications as follows. A single colony was cultured overnight at 30°C in 1.5 mL of tryptic soy broth (Difco). Cells were harvested by centrifugation at 9000 × *g* for 5 min, followed by washing with 1 mL of distilled water and subsequent collection by centrifugation. The cell pellet was resuspended in 300 µL of EDTA‐saline solution (0.01 M EDTA, 0.15 M NaCl, pH 8.0) and thoroughly vortexed. The cell pellet was resuspended in 300 µL of EDTA‐saline solution (0.01 M EDTA, 0.15 M NaCl, pH 8.0) and vortexed thoroughly. Cell lysis and RNA elimination were initiated in succession and partially simultaneously by adding 50 µL of 110 mg/mL lysozyme (SERVA) and 10 µL of 20 mg/mL RNase A (Thermo Fisher Scientific), mixing by vortexing, and incubating at 37°C for 2 h with vortexing every 15 min. After incubation, 160 µL of 20% SDS and 20 µL of 5 mg/ml proteinase K (Merck) were added, followed by vortexing and incubation at 65°C for 30 min. Next, 0.5 volume of 5 M NaCl was added and briefly mixed. An equal volume of phenol (1:1, v/v) was added, vortexed, and centrifuged at 12,000 × *g* for 15 min. The upper aqueous phase was transferred to a new tube, and this extraction step was repeated once more. Then, 2 µL of 20 mg/mL RNase A was added and incubated for 15 min, followed by a final phenol extraction. The upper phase was transferred to a new tube. To precipitate the DNA, 0.1 volume of 3 M sodium acetate (pH 5.2) was added and mixed gently, followed by the addition of 2 volumes of cold absolute ethanol. The mixture was incubated at −20°C for 2 h and then centrifuged for 10 min to collect the DNA pellet. The DNA pellet was washed with cold 70% ethanol, air‐dried, and resuspended in 20 µl of RNase‐ and DNase‐free water. DNA quality and quantity were measured using a Nanodrop spectrophotometer.

### 2.4 Sequencing

Bacterial DNA samples (1.0 µg each) were sent for whole genome sequencing to BGI Hong Kong Tech Solution NGS Lab via Bangkok Genomics Innovation Co. Ltd. (Thailand). DNA quantity and quality were verified using microplate reading and gel electrophoresis before library preparation. The DNA libraries were then prepared using the short‐insert library method and sequenced with DNBseq technology at a PE150 read length generating 4 million reads per sample. Data filtering, including the removal of adaptor sequences and low‐quality reads, was performed by the company.

### 2.5 Data Analysis

Assemblies were created using NF‐core Bacass (Peltzer 2023) v2.2.0 under short read mode. Briefly, the reads are assessed for quality using FastQC v0.12.0 (https://www.bioinformatics.babraham.ac.uk/projects/fastqc/) and trimmed using FastP (Chen et al. 2018) v0.23.4. Clean and high quality reads are assembled using Unicycler (Wick et al. 2017) v0.5.0 and the resulting contigs were checked and filtered for taxonomic assignment using Kraken2 (Wood et al. 2019) v2.1.3.

Genome assemblies have been deposited in National Library of Medicine (NCBI) GenBank under the BioProject PRJNA1264975.

Taxonomic classification of genomes was calculated using digital DNA:DNA hybridisation with the Genome to Genome Distance Calculator (GGDC) (Meier‐Kolthoff et al. 2022) using reference genomes *L. garvieae* GCA_016026695, *L. petauri* GCA_023499275 and *L. formosensis* GCA_037892375. Relatedness of bacterial genomes was tested using Sourmash (Titus Brown and Irber 2016) on average nucleotide identity (ANI) mode and heatmaps were created in R v4.3.2 (R Core Team 2023) using Pheatmap (https://github.com/raivokolde/pheatmap).

The genomes were parsed for genotypic AMR using the AMR + + and the MegaRes3 database (Bonin et al. 2023). All 132 nonredundant sequences reported by Lin et al (Lin et al. 2023) covering adhesins (*psaA, pavA, eno*), capsule‐biosynthesis genes (cps cluster), iron‐uptake systems (*fhu and feo* operons), sortase‐anchored LPXTG proteins, and other confirmed or putative virulence factors were downloaded from GenBank. The final fasta library was formatted into an Abricate database with the ‐‐setupdb command. Gene presence or absence was determined using Abricate v1.0.0 (https://github.com/tseemann/abricate).

Presence of prophage regions was determined using three tools; geNomad v1.8.0 (Camargo et al. 2023), PhageBoost v0.1.7 (Sirén et al. 2021) and VIBRANT v1.2.1 (Kieft et al. 2020). These tools were selected as they use different combinations of reference databases, biological features and machine learning to ensure robust prophage prediction. The predictions were then consolidated by selecting the start and end location of the prophage region which resulted in the longest possible region, using Biopython (Cock et al. 2009). The resulting sequences were processed using CheckV v1.0.3 (Nayfach et al. 2021), retaining only sequences identified as ‘medium quality’ or higher. Additionally, where host contamination was identified by CheckV, the trimmed sequence was kept. Sequences from the same host were dereplicated using CheckV supporting code. Finally, the sequences were annotated first using Pharokka (Bouras et al. 2023) v1.7.5. The output from Pharokka was subsequently used an input for phold v0.2.0 (https://github.com/gbouras13/phold) as phold outperforms Pharokka especially when considering less characterised phage.

Taxonomic assignment was completed using taxmyPHAGE (Millard 2024) and proteomic trees were generated using Viral Proteomic Tree (ViPTree). Genomic level comparisons of the prophage were performed using CheckV supporting ANI and clustering code, default clustering cutoffs of 95% ANI over 85% alignment fraction were used as described by Roux et al (Roux et al. 2019). Genomic organisation and protein clustering was performed using LoVis4u v0.1.1 (Egorov and Atkinson 2025), loci were reorientated, and functional annotation was performed for defence, anti‐defence, virulence and AMR genes. Prophage were considered ‘intact’ if they contained an integrase, structural proteins and lysis modules in the conserved gene order as assessed by manual curation (Ridgway et al. 2024; Canchaya et al. 2003). Finally, the termini of prophage regions of interest were investigated using PhageTermVirome v4.1 (Garneau et al. 2021).

## 3 Results

### 3.1 Genome Assembly

The quality control data revealed a mean Phred quality of 39 and *de novo* assembled following the Bacass (Peltzer 2023) short read pipeline to approximately 2.0 Mbp (min 2.0 Mbp max 2.3 Mbp) with a mean N50 of 756 Kbp. Based on Kraken2 (Wood et al. 2019) the final assemblies were classified as *Lactococcus* species. Further taxonomic assignment was generated through Digital DNA:DNA Hybridization (dDDH) which yielded a species level classification with probability scores ≥ 93% to reference species *L. garvieae* GCA_016026695 and *L. petauri* GCA_023499275, corresponding to 3 and 12 isolates, respectively (Table 1).

### 3.2 Genome Comparison

**Figure 1:**
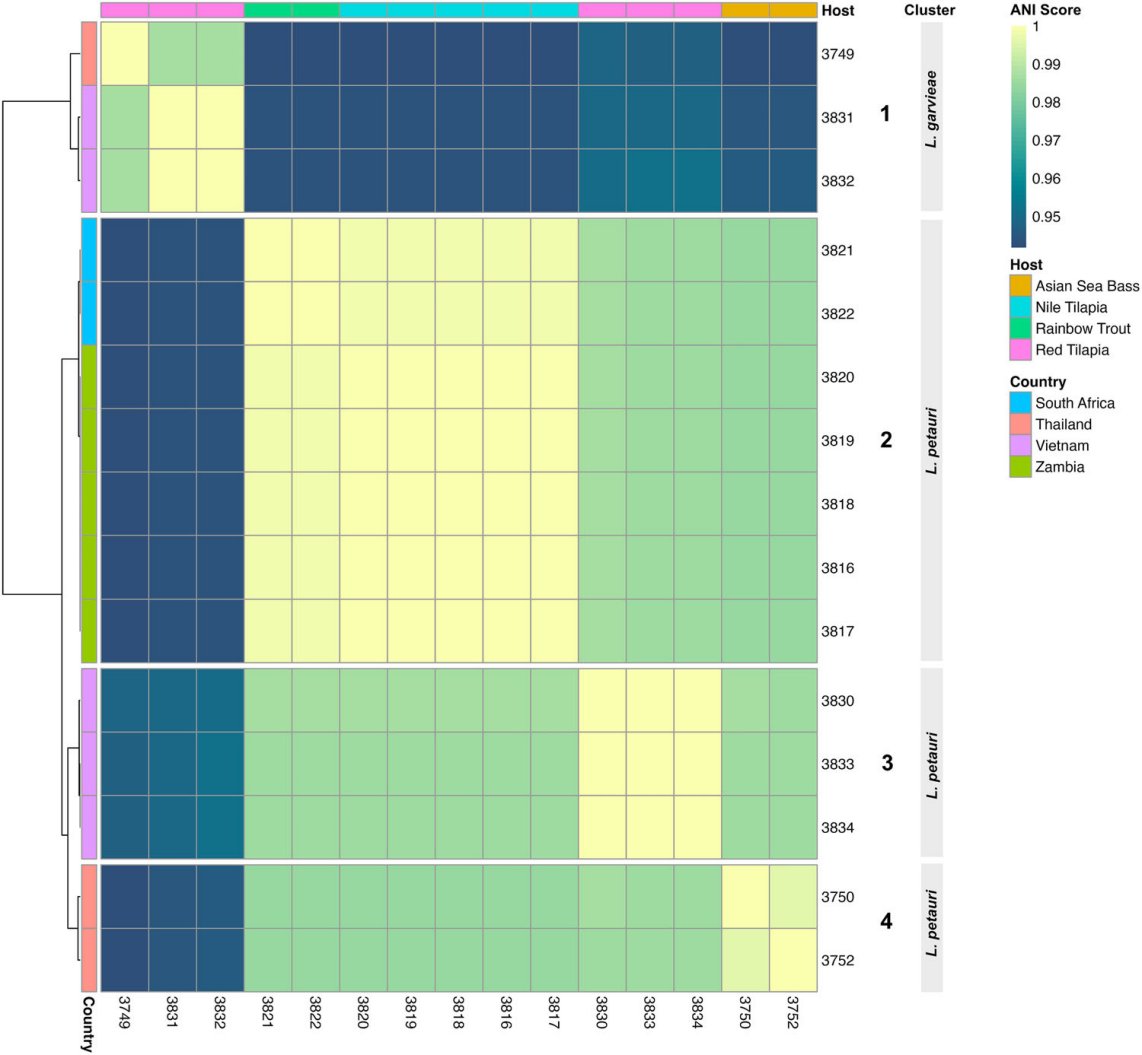
Average Nucleotide Identity comparison of all isolates. ANI scores were calculated based on whole‐genome sequencing data of 15 Lactococcus isolates from fish hosts (Asian seabass, Nile tilapia, rainbow trout, and red tilapia) across four countries (South Africa, Thailand, Vietnam, and Zambia). The heatmap presents pairwise ANI comparisons between isolates, with lighter yellow‐green shades indicating higher similarity ( ≥ 0.99 ANI), and darker blue indicating lower genomic similarity ( ≤ 0.95 ANI). Isolates are grouped into four distinct clusters representing Lactococcus species (*L. garvieae and L. petauri*), as indicated on the right. Clustering was based on hierarchical analysis of ANI values. Host species and country of origin for each isolate are indicated by coloured annotations at the top and left side of the heatmap, respectively.

Based on average nucleotide identity scores (Figure 1), the isolates seem to form two clusters based on species classification. Cluster 1 is the *L. garvieae* isolates, where clusters 2, 3, and 4 are all *L. petauri* where they split into country of origin. Cluster 2 contains isolates from Zambia and South Africa whereas cluster 3 is Vietnamese isolates and cluster 4 is Thai isolates.

### 3.3 AMR

All isolates showed multi‐drug genotypic resistance with the presence of *mdtA* (drug, biocide and metal RND efflux pumps) and *lsaD* (multi‐drug ABC efflux pumps). Two isolates (3833 and 3834) had *mefA* (MLS erythromycin resistance MFS efflux pumps) and *msrD* (MLS erythromycin resistance ABC efflux pumps). Only two isolates (3821 and 3822) had plasmid borne tetracycline genotypic resistance with the presence of *tetL* (accession NF012176.0) and three isolates (3750, 3821, and 3822) had *tetS*. Also, two isolates (3833 and 3834) had *lnuC* (lincosamide) genotypic resistance (Supp Table 1). Overall, the isolates could be ascribed to four resistance profiles, the most common being MLS efflux pumps (Table 2).

**Table 2:** Antimicrobial resistance genotypic profiles for the isolates.

ID	mdtA	lsaD	lnuC	mefA	msrD	tetL	tetS	Species	Country
3749	•	•						*L. garvieae*	Thailand
3750	•	•					•	*L. petauri*	Thailand
3752	•	•						*L. petauri*	Thailand
3816	•	•						*L. petauri*	Zambia
3817	•	•						*L. petauri*	Zambia
3818	•	•						*L. petauri*	Zambia
3819	•	•						*L. petauri*	Zambia
3820	•	•						*L. petauri*	Zambia
3821	•	•				•	•	*L. petauri*	S. Africa
3822	•	•				•	•	*L. petauri*	S. Africa
3830	•	•						*L. petauri*	Vietnam
3831	•	•						*L. garvieae*	Vietnam
3832	•	•						*L. garvieae*	Vietnam
3833	•	•	•	•	•			*L. petauri*	Vietnam
3834	•	•	•	•	•			*L. petauri*	Vietnam

*Note:* Gene names and linked function*: mdtA* Drug and biocide and metal RND efflux pumps, *lsaD* Multi‐drug ABC efflux pumps, *lnuC* Lincosamide nucleotidyltransferases, *mefA* MLS erythromycin resistance MFS efflux pumps, *msrD* MLS erythromycin resistance MFS efflux pumps, *tetL* Tetracycline resistance MFS efflux pumps, and *tetS* Tetracycline resistance ribosomal protection proteins. Full data in Table S1.

The Lactococcus strains were tested for susceptibility to eight antimicrobial agents. All 15 isolates exhibited resistance to nalidixic acid (NA), 12 to trimethoprim (TR), and 11 to sulfamethoxazole (SMX). Additionally, seven isolates were resistant to ciprofloxacin (CIP) and oxacillin (OX) (Table 3).

**Table 3:** Antimicrobial susceptibility profiles of lactococcus spp. to eight antimicrobials.

Antimicrobial classes	Antimicrobial agents	Disk content (µg)	Zone diameter			Resistance profile															
						Thailand			Zambia					South Africa		Vietnam					*S. aureus*
			S	I	R	3749	3750	3752	3816	3817	3818	3819	3820	3821	3822	3830	3831	3832	3833	3834	1020
Tetracycline	Oxytetracycline (OT)	30	≥ 19	15–18	≤ 14	R	R	S	S	S	S	S	S	R	R	R	S	S	R	S	S
Macrolides	Erythromycin (ETM)	15	≥ 23	14–22	≤ 13	R	S	S	S	S	S	S	S	S	I	R	S	R	R	R	S
Fluoroquinolone	Ciprofloxacin (CIP)	10	≥ 21	16–20	≤ 15	R	S	S	S	S	I	S	I	S	R	R	R	R	R	R	S
quinolone antibiotic	Nalidixic acid (NA)	30	≥ 19	14–18	≤ 13	R	R	R	R	R	R	R	R	R	R	R	R	R	R	R	S
Sulfonamides	Sulfamethoxazole (SX)	25	≥ 25	21–24	≤ 20	R	R	I	R	R	R	R	R	S	R	R	I	R	I	R	S
	Trimethoprim (TR)	30	≥ 16	11–15	≤ 10	R	R	S	R	R	R	S	S	R	R	R	R	R	R	R	R
β‐lactam	Amoxicillin (AML)	30	≥ 18	14−17	≤ 13	R	S	S	S	S	S	S	S	S	S	R	S	S	R	S	I
	Oxacillin (OX)	5	≥ 20	—	≤ 20	R	R	S	S	S	S	S	R	S	R	R	S	R	R	S	R
MAR index						1	0.625	0.125	0.375	0.375	0.375	0.25	0.375	0.375	0.75	1	0.375	0.75	0.875	0.625	0.25

Abbreviations: I = intermediate, R = resistant, S = susceptible.

### 3.4 Virulence Genotype

Based on a cutoff of ≥ 95 identity, all isolates had the three haemolysin genes, the nine iron uptake genes and adhesion genes (enolase, *pavA* and *psaA*) but only three isolates (3749, 3831 and 3832) had adhesin. All isolates had GAPDH, NADH oxidase, phosphoglucomutase, superoxide dismutase and collagenase genes. Only two isolates had a complete capsule operon of all 12 loci (3830, 3832), while two isolates 3833 and 3834 had 11 genes from the operon, each missing the 1p transposase. All isolates had *srtA* and LPxTG‐1, and nine isolates (3861, 3817, 3818, 3819, 3820, 3821, 3822, 3831 and 3832 had LPxTG‐3 and LPxTG‐6 while 3749 only had LPxTG‐6. Only two isolates (3821 and 3832) had LPxTG‐2 and LPxTG‐4 (Table S2).

### 3.5 Prophage

**Figure 2:**
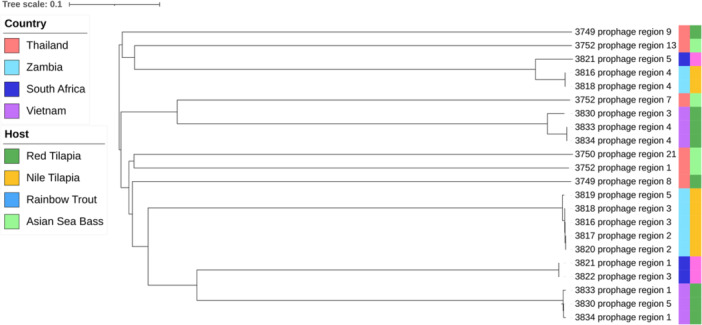
Prophage proteomic phylogenetic tree. The tree represents evolutionary relationships among prophage identified within 15 Lactococcus isolates obtained from aquaculture outbreaks in South Africa, Thailand, Vietnam, and Zambia. The tree was constructed using VipTree protein alignments of predicted prophage regions, with branch lengths indicating evolutionary distances (scale bar represents 1 substitution per 10 bases). Each tip label indicates the isolate identifier and corresponding prophage region. The country of origin and fish host species for each isolate are represented by coloured boxes on the right. This analysis demonstrates the genetic diversity and distribution of prophages within Lactococcus populations, highlighting their potential role in genetic variability and phage resistance mechanisms in aquaculture environments.

A total of 144 putative prophage regions were identified in the isolates, 22 of which were retained after filtering with CheckV. Only two (2/15, 13%) *Lactococcus* genomes did not contain any prophage regions whereas 1‐3 prophage regions were identified in the remaining genomes (13/15, 87%). The prophage regions were between 13 and 69 kbp in length and contained between 29 and 129 coding sequences. taxmyPHAGE assigned all the prophage to novel genera, however, 3830 prophage 5, 3833 prophage 1, and 3834 prophage 1 were taxonomically similar to *Siphovirus*‐like phage genus *Piorkowskivirus*. Comparison of the regions using a proteomic tree did not identify clustering based on host species or country of origin (Figure 2). Additionally, when viewed on a proteomic tree with 3,399 sequences from the ViPTree viral database, the prophage were not closely related. However, they did all fall within the area of the tree with viruses of *Bacillota* (previously *Firmicutes*; Figure S1). The majority (59%;13/22) of the prophage were similar to other *Lactococcus* phage. The remaining regions clustered with phage that infect *Thermoanaerobacterium*, *Streptococcus*, *Enterococcus*, and *Lactobacillus*.

As the prophage regions could not be taxonomically classified, the genomic diversity of the prophage was investigated using pairwise ANI, followed by clustering using thresholds of 95% ANI over 85% alignment fraction, this identified 11 putative virus operational taxonomic units (vOTUs) (Supporting Information S1: Figure S2). Similarly, when the annotated prophage were visualised with LoVis4u, protein clustering supported 11 vOTUs (Supporting Information S1: Figure S3). This revealed that the proteins within prophage regions were highly conserved within vOTUs. This is particularly interesting for prophage identified in the genomes of *L. petauri* 3816‐20 as these samples were collected from infected Nile tilapia found in Lake Kariba across a period of almost 2 years suggesting the persistence of *L. petauri* within the tilapia population over this period.

**Figure 3:**
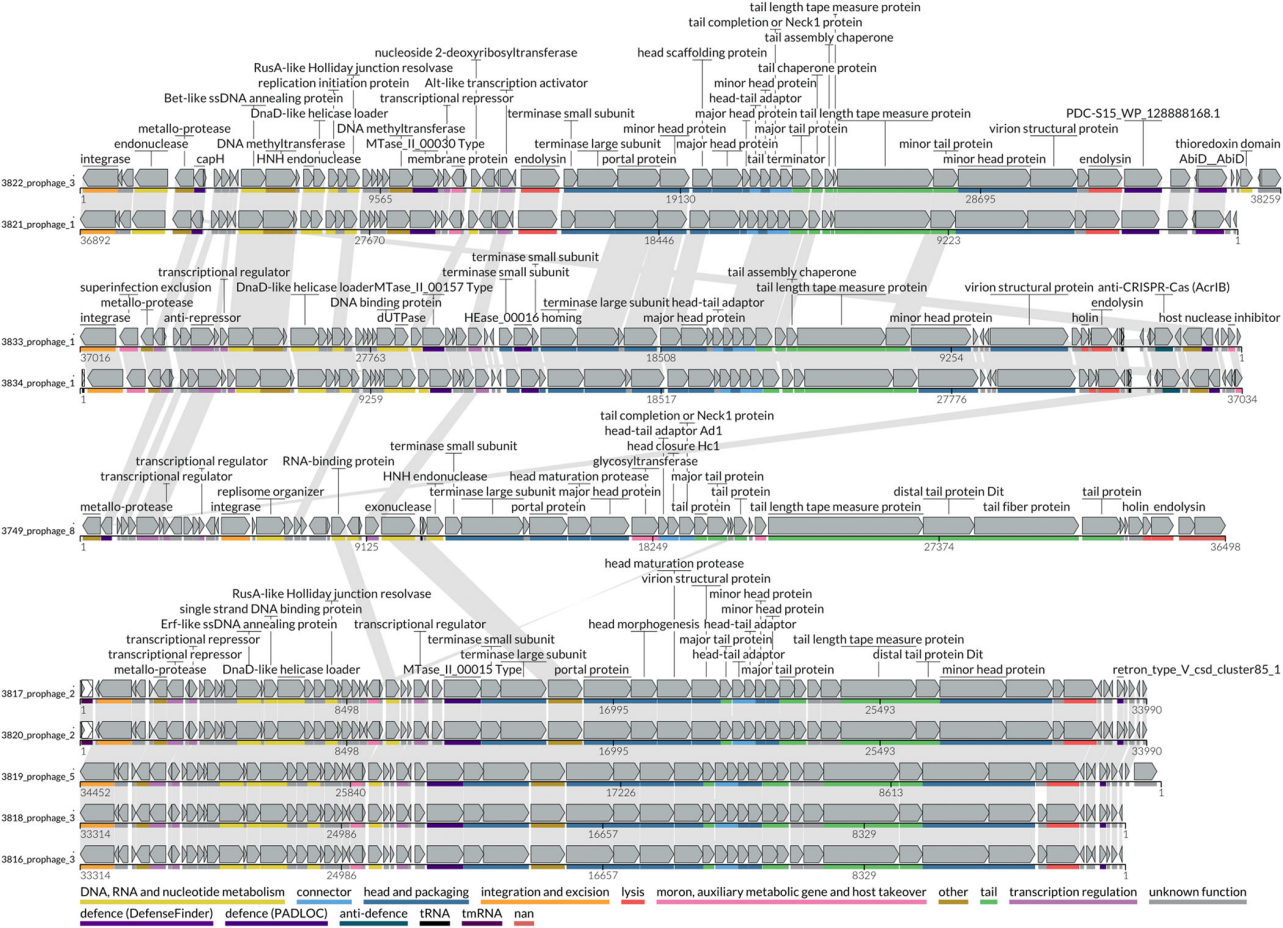
Genomic organisation of the identified intact prophage regions. Linear representations illustrate the genomic architecture of unique prophage regions detected within the Lactococcus isolates. Genes are represented by arrows indicating the predicted open reading frames (ORFs), colour‐coded according to functional annotation categories: DNA, RNA, and nucleotide metabolism; connector proteins; head and packaging proteins; integration and excision machinery; lysis proteins; moron, auxiliary metabolic genes, and host takeover proteins; tail proteins; transcriptional regulators; and defence related elements. Genes of unknown function or hypothetical proteins are depicted in grey. Selected gene functions and annotations are highlighted above the corresponding ORFs for clarity.

When the prophage regions were interrogated for the presence of AMR and virulence determinants, a number of genes were identified. However, despite the comprehensive methods used to identify prophage regions, only 45% (10/22) were found to contain protein sequences indicative of an intact prophage region. No virulence or AMR genes were identified within an intact prophage region (Figure 3). Despite this, several defence genes were identified associated with intact prophage regions, this would be an important consideration were bacteriophage treatment investigated as an alternative to antibiotics. Specifically, a coding sequence identified as AbiD was found in two prophage regions. Abi systems have been previously reported on lactococcal plasmids (Mills et al. 2006). To confirm that AbiD was within the prophage region, PhageTermVirome was used to identify the termini of prophage 3822 prophage 3 and 3821 prophage 1. The termini of 3822 prophage 3 could not be determined, however the termini of 3821 prophage 1 were identified and included the coding sequence of AbiD.

## 4 Discussion

This study characterised the genomes of 15 *Lactococcus* strains isolated from piscine disease outbreaks in Asia and Africa. This revealed the presence of up to five AMR genes and three novel prophage regions per strain. Virulence gene characterisation found that only *L. garvieae* isolates carried the adhesin gene *adh* which is critical for bacterial colonisation. Although the prophage regions did not harbour any known virulence or AMR genes, they did contain several genes encoding phage defence systems such as (AbiD and R‐M systems). This information is important when considering the potential development of phage therapy to control piscine disease.

Interestingly, most isolates from cases of lactococcosis in the present study were not *L. garvieae* (*n* = 3)—the most commonly recognised aetiological agent, but *L. petauri* (*n* = 12). The predominant detection of *L. petauri* in this study, supported by recent reports (Egger et al. 2023; Vela et al. 2024), suggesting that this species may have previously been either overlooked, or misidentified as *L. garvieae*, due to its high phenotypic and 16S rRNA gene sequence similarity (Heckman et al. 2024). These findings underscore the importance of implementing both active and retrospective surveillance strategies for *Lactococcus* spp. in fish populations to improve species‐level identification and enhance disease management strategies. Only three isolates (3749, 3831, and 3832) had the adhesin gene (*adh*), which seems specific to the *L. garvieae* isolates. Adhesins are necessary for the ability of the bacteria to bind to the host cell surface and are required for colonisation and pathogenesis. Despite the essentiality of these loci, it has been documented that some strains of *L. garvieae* do not possess all three loci *adh, pav* and *psaA (*Ture and Altinok 2016 *)* and these variations may hint to host specificity (Klemm and Schembri 2000).

### 4.1 Sortase Proteins Linked to Increased Virulence

Most of the isolates investigated possessed multiple sortase‐anchored proteins, however, the low virulence isolate, 3749 only had LPxTG‐6 and isolates 3821 and the high virulence isolate 3832 had LPxTG‐2 and LPxTG‐4. These sortase anchored proteins in particular are highly associated with the iron uptake operon, adhesion and haemolysis (Lin et al. 2023). This may aid in colonisation and stress resistance, enabling adaptation and survival in nutrient‐poor or hostile environments. The LPxTG‐6 subgroup is unique to isolates 3749, 3821, and 3822 which came from two different locations. There is a dearth of information on the specific functional implications of different sortase anchored protein variants, but due to the high co‐occurrence of LPxTG‐6 to the iron uptake operon, function could be suggested to play an important role in nutrient uptake, whereas LPxTG‐4 seems to be more associated with *psaA*, which could hint at a roll in initial colonisation (Lin et al. 2023).

Only isolates 3833 and 3834 had an incomplete complement of 12 genes from the capsule operon, each missing the 1p transposase. The feature of genes encoding transposases or phage‐related proteins within a capsule operon may suggest evidence of transposition or horizontal gene transfer. It is likely they alter production of the capsule related proteins rather than complete disruption, serving as a method of immune evasion. This has been documented in other Gram positive bacteria, where the transposase loci in *Streptococcus pneumoniae* differentiates between serotypes in serogroup 12 (Bentley et al. 2006).

### 4.2 Development of AMR to Commonly Used Antibiotics

All isolates processed the efflux pumps *mdtA* which are associated with macrolide resistance, and *lsaD* associated with lincosamide resistance. These resistances are commonly found generally in Gram positive bacteria and would not be of cause for concern in first line treatment of infections. However, two isolates showed genotypic resistance to erythromycin (*mefA* and *msrD*). While not uncommon in Gram positive bacteria, erythromycin has been used in prophylactic feed based medication in aquaculture, which has driven the selective pressure for resistant strains of *Lactococcus* (Soltani et al. 2021). Finally, three isolates showed genotypic resistance to tetracyclines with the presence of *tetS* and two additionally showed plasmid‐borne tetracycline resistance from *tetL*. This resistance is also prevalent in isolates from aquacultural systems, and has been detected more frequently since the increased use of tetracycline for treatment of lactococcosis from 2014 (Torres‐Corral and Santos 2022). There might be a greater concern from the plasmid‐borne nature of *tetS* and the potential of its ability to transfer if stock is imported from different brood stock providers.

For the phenotypic sensitivity testing, the antimicrobial panel was selected to reflect classes commonly used or detected in aquaculture rather than to predict clinical therapy in *Lactococcus*. Quinolones, sulfonamides/trimethoprim, β‐lactams, macrolides, and tetracyclines are among the most frequently applied in fish farming, and nalidixic acid and ciprofloxacin therefore served as quinolone markers, trimethoprim–sulfamethoxazole as a folate pathway inhibitor, and oxacillin as a β‐lactam indicator. Using this panel, all 15 isolates were resistant to nalidixic acid, 12 to trimethoprim, 11 to sulfamethoxazole, and seven to ciprofloxacin and oxacillin.

These results did not directly correspond to the genotypic determinants detected (*mefA, lsaD, lnuC, tetL, tetS, mdtA*), which primarily confer resistance to macrolides, lincosamides, and tetracyclines. Quinolone resistance in streptococci and lactococci is usually driven by mutations in *gyrA* or *parC* or by nonspecific efflux, rather than transferable ARGs. Nalidixic acid also has intrinsically poor activity against Gram‐positive cocci, explaining the uniform resistance observed. Resistance to trimethoprim and sulfamethoxazole may reflect folate pathway variants, but false resistance is also common on non‐thymidine‐controlled media. Oxacillin, while included as a β‐lactam proxy, is validated only for *Staphylococcus* and not clinically interpretable in *Lactococcus*. Overall, the phenotypes largely represent aquaculture‐relevant selection pressures, while the ARG profile captures transferable mechanisms of greater clinical concern.

In the context of Thai aquaculture, where trimethoprim is one of the few antibiotics permitted for use, these findings suggest that trimethoprim may have limited efficacy for managing Lactococcus infections in affected farms. The MAR indices of the Lactococcus isolates ranged from 0.12 to 1.0. Isolates from Thailand and Vietnam tended to display higher MAR values compared with those from Zambia and South Africa, which may also reflect differences in host fish species. In this study, isolates recovered from red tilapia generally exhibited higher MAR indices than those from other fish hosts. Although broader sampling across additional regions and fish species is needed to confirm these trends, antibiotic resistance in Lactococcus has also been reported in other aquaculture systems, including in cultured yellowtail, amberjack, and kingfish in Japan (Kawanishi et al. 2005) and in rainbow trout (*Oncorhynchus mykiss*) (Raissy and Ansari 2011).

### 4.3 Identification of Phage Defence Mechanisms

In the present study, the incidence of prophage within the genome sequences of *Lactococcus* spp. from global aquaculture outbreaks was investigated. This identified 22 regions, 10 of which were considered intact. The rapid development of AMR has focused attention on alternative treatments such as bacteriophage therapy, bacterial viruses that infect and replicate within bacterial cells, as an alternative to antibiotics. Only one study has attempted phage therapy against *L. garvieae* infections in yellowtail fish, which reported significant reductions in mortality in fish treated with phage either injected peritoneally, or through feed (Nakai et al. 1999). However, timing of phage delivery was critical, with mortality appreciably higher when treatment was delayed to 24 h after bacterial challenge.

Despite promising results, there are some limitations of bacteriophage therapy, namely the development of resistance which has been discussed in detail elsewhere (Labrie et al. 2010). Restriction‐modification (R‐M) systems are ubiquitous among bacterial chromosomes and plasmids, these function by degrading DNA while protecting self‐DNA with modification (Bernheim and Sorek 2020). Coding sequences identified as type II DNA methyltransferases and endonucleases were present in multiple prophage regions. Although involved in other phage processes, these are thought to protect the prophage from host R‐M systems during the lytic phase (Murphy et al. 2013).

One mechanism by which bacteria can be resistant to bacteriophage infection is related to prophage, temperate bacterial viruses which are incorporated in the bacterial genome which can confer phenotypic advantages in environmental niches. These advantages may include increased resistance to acid or antimicrobials, enhanced biofilm production, higher growth rates, and modulation of eukaryotic host immune response (Cieślik et al. 2021). Prophage‐encoded proteins of *Streptococcus mitis* (Bensing et al. 2001) and *Enterococcus faecalis (*Matos et al. 2013 *)* facilitate adhesion to human platelets which is crucial for the development of endocarditis, and may be relevant to *L. garvieae* pathophysiology. Temperate phage are also associated with horizontal gene transfer, via transduction, and conferring superinfection immunity to closely related phage. In the present study, AbiD was identified within the one of the prophage regions. Abortive infection (Abi) systems are diverse in *Lactococcus lactis* and generally function by causing death of the bacterial host (Chopin et al. 2005), consequently, the presence of Abi systems may have unexpected effects if bacteriophage were used to treat these infections.

Typically, for these reasons, temperate phage are considered unsuitable for use as direct therapeutic agents. However, in cases where strictly‐lytic phage are rare or impossible to find, prophage can be used as the basis for developing effective treatments (Mondal et al. 2020). Most commonly, this includes identifying sequences encoding hydrolytic enzymes (usually endolysins) phage use to escape from infected cells after replication, then expressing and purifying the proteins. Endolysins are particularly effective against Gram positive bacterial pathogens which lack an outer membrane to defend their cell wall from digestion (Fischetti 2010). Lytic and temperate phage which infect *L. garvieae* have been isolated (Wood et al. 2019; Meier‐Kolthoff et al. 2022). However, only minimal data is available on the lytic spectrum of these phage – which indicates a very limited host range ‐ so it is difficult to determine if they would be useful against the broad diversity of *L. garvieae* strains causing disease.

Although tilapia is native to Africa, it has been domesticated and cultivated in over 140 countries worldwide. Seed production and aquaculture technologies have advanced significantly in Asia. African countries have a long history of importing tilapia seed from Asian nations. Movement of live fish, which may carry pathogens through subclinical infections for aquaculture, could contribute to the translocation of these pathogens, similar to the dynamics observed with viruses such as TiLV and ISKNV (Ramírez‐Paredes et al. 2021; Dong et al. 2017). The genomic analysis in this study suggest that the African isolates and Asian isolates form different unique clusters, suggesting their diversification in genetic evolution. Nonetheless, this study provides some fundamental knowledge of genomic characterization of the piscine *Lactococcus* spp., which is useful for further study on distribution and tracking genomic epidemiology as reported earlier with other pathogens (Thawornwattana et al. 2021).

In summary, the predominance of *L. petauri* among isolates from lactococcosis outbreaks in fish farms in South Africa and Southeast Asia could indicate a potential shift in the etiological landscape of this disease. Comparative genomic analyses revealed distinct differences between *L. petauri* and *L. garvieae* in terms of virulence‐associated factors, AMR genes, and prophage content. These findings underscore the need for enhanced taxonomic resolution in diagnostic protocols and reinforce the importance of ongoing genomic surveillance. Adaptation of current disease management and treatment strategies is warranted to address the emerging role of *L. petauri* in aquaculture, particularly in light of its potential for AMR and horizontal gene transfer.

## Author Contributions

**Adam M. Blanchard:** methodology, software, data curation, investigation, validation, formal analysis, visualisation, writing – original draft, writing – review and editing. **Bailey Secker:** software, formal analysis, visualisation, writing – original draft, writing – review and editing. **Robert J. Atterbury:** methodology, software, investigation, writing – original draft, writing – review and editing. **Samantha J. Windle:** software, data curation, formal analysis, writing – original draft, writing – review and editing. **Ha Thanh Dong:** conceptualization, methodology, investigation, validation, project administration, resources, writing – original draft, writing – review and editing. **Janchai Wongkaew:** methodology, data curation, investigation, validation, formal analysis, resources. **Le Thanh Dien:** conceptualization, methodology, investigation, formal analysis, writing – original draft, writing – review and editing. **David Huchzermeyer:** conceptualization, methodology, investigation, writing – original draft, writing – review and editing. **Bernard Mudenda Hang'ombe:** conceptualization, methodology, data curation, writing – original draft, writing – review and editing. **Saengchan Senapin:** conceptualization, methodology, data curation, investigation, formal analysis, project administration, writing – original draft, writing – review and editing.

## Conflicts of Interest

The authors declare no conflicts of interest.

## Supporting information

Supplementary Table 1 Antimicrobial resistance genes.

Supplementary Table 2 Virulence Associated Genes.

Supp Figures.

## Data Availability

Genome assemblies have been deposited in National Library of Medicine (NCBI) GenBank under the BioProject PRJNA1264975.
