# Supplementary material for: Comparative Genomics of Lactococcus spp. From Global Aquaculture Outbreaks Reveals Virulence Determinants, Antibiotic Resistance, and Phage Defence Mechanisms
Source: Microbiologyopen. 2025 Nov 14;14(6):e70147. doi: 10.1002/mbo3.70147 (PMC12616882; doi:10.1002/mbo3.70147)
Supplement: Supplementary file 3 — Supp Figures. [file MBO3-14-e70147-s003.docx]

**
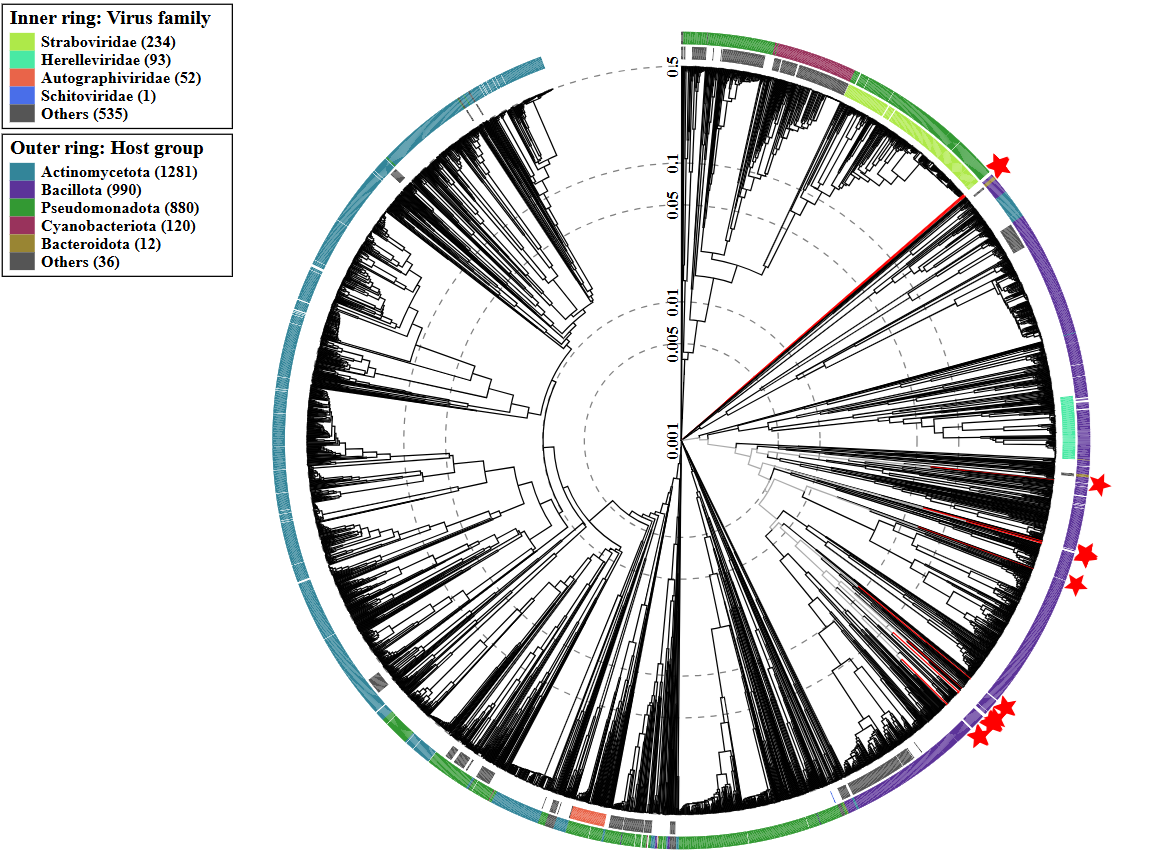
Supplementary Figures**

**Supplementary Figure 1 Proteomic phylogenetic tree generated using VipTree.** VipTree was used to generate a proteomic tree using 22 prophage regions identified as part of the present study in addition to 3399 prokaryotic dsDNA viruses.

**
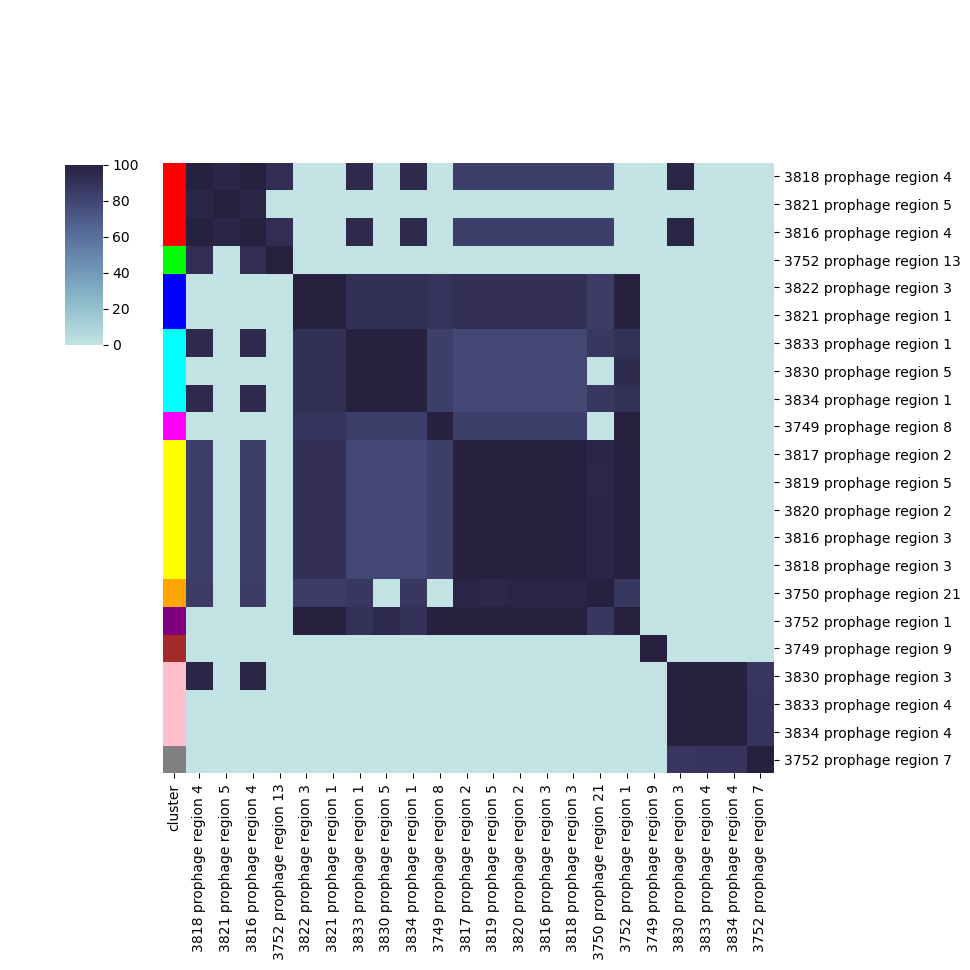
**

**Supplementary Figure 2 Genomic level comparisons of the prophage using average nucleotide identity (ANI).** Clustering shown on the left was performed using cutoffs of 95% ANI over 85% alignment fraction. ANI and clustering was performed using CheckV supporting code.

**
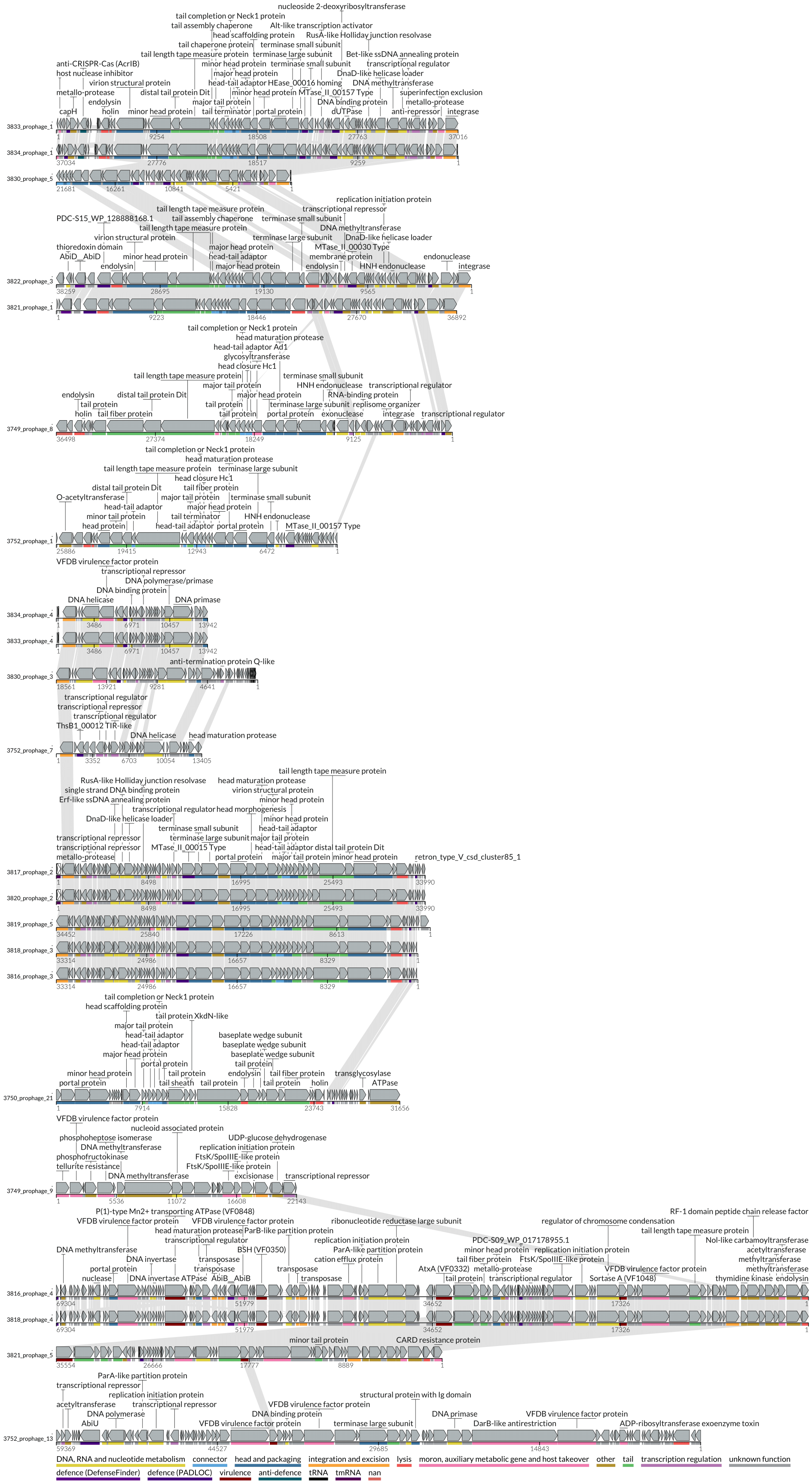
**

**Supplementary Figure 3 Genomic organisation of the 22 prophage regions identified as part of the present study.** The genomes are reorientated and homology between coding sequences is shown. Coding sequences are coloured based on presumed function.
